# An Exploratory Study on Sleep Procrastination: Bedtime vs. While-in-Bed Procrastination

**DOI:** 10.3390/ijerph17165892

**Published:** 2020-08-13

**Authors:** Paula Magalhães, Vânia Cruz, Sara Teixeira, Sónia Fuentes, Pedro Rosário

**Affiliations:** 1 Escola de Psicologia, Universidade do Minho, 4710-057 Braga, Portugal; vaaniacruz@hotmail.com (V.C.); sarateixeira.psi@gmail.com (S.T.); prosario@psi.uminho.pt (P.R.); 2 Facultad de Educación, Universidad Central de Chile, Santiago 530-598, Chile; sofu2028@gmail.com

**Keywords:** sleep insufficiency, sleep procrastination, bedtime procrastination, while-in-bed procrastination, adolescents

## Abstract

Sleep Procrastination literature has focused on the behaviors individuals engage in before going to bed (Bedtime Procrastination) but not on the behaviors individuals engage in after going to bed (While-in-Bed Procrastination). The main goal of this study is to explore whether this While-in-Bed procrastination is a novel phenomenon that adds to the Sleep Procrastination literature. The study was conducted online with 400 high school students (*M_age_* = 16.56; 139 males) recruited through personal contacts and social media. The Bedtime procrastination scale was adapted and validated for this sample, whereas the While-in-Bed Procrastination scale was developed for this study. Data show a low correlation (r = 0.158 **) between Bedtime and While-in-Bed Procrastination scales, suggesting that Sleep Procrastination may be composed of the two facets. Additionally, results showed that more Bedtime Procrastination was related to later waking time and later dinnertime hours, whereas more While-in-Bed Procrastination was linked to being male, later desired time to sleep, and earlier dinnertime hour. Findings indicate that solely assessing Bedtime Procrastination as representing the procrastination of Sleep is limited and overlooks a significant part of this behavior. This exploratory study adds a new perspective to the literature by stressing the role of While-in-Bed Procrastination, thus opening new research pathways.

## 1. Introduction

Sleep has a key role in maintaining our body homeostasis and overall function. However, about 70% of adolescents are sleeping less than 8 h per night, when the recommended amount of sleep for teenagers is 8 to 10 h per night [1,2]. In fact, about 45% of adolescents between the 6th and 12th grades are affected by sleep insufficiency [3,4]. Considering sleep’s key role in an individual’s health and overall wellbeing, it comes as no surprise that sleep insufficiency has been recognized as a major health issue [5].

Adolescence is a period of transition from childhood to adulthood, which leads to transformations at a hormonal, somatic, neurological, and behavioral level. These processes occur at the same time as maturational sleep mechanisms and other complex processes in the body [6]. Literature has shown that sleep plays a crucial part in brain maturation, particularly when the brain suffers significant changes [7,8,9,10]. During adolescence, sleep behavior and routines change. This change is the result of an intertwined set of biological and social transformations that conjointly contribute to sleep deprivation among adolescents [7,11]. For example, this developmental period is characterized by changes in the sleep architecture, evidenced in EEG data [7,11]. It is also characterized by a shift in an individual’s chronotype towards eveningness that appears to be unrelated with contextual or cultural factors but may be due to changes in the circadian and homeostatic processes [7,11].

Sleep insufficiency in adolescence is linked to risk-taking behavior, such as high use of alcohol, tobacco, and marijuana; high-risk sexual behaviors; and traffic accidents e.g., [7,12,13]. In addition, sleep insufficiency constitutes a risk for obesity, depression, and suicide attempts e.g., [7,14,15,16,17]. It affects mood and emotion regulation [18] and is related to low emotional competence. Furthermore, academic performance of adolescents is affected by this condition. Sleep deprivation leads to emotional problems and injuries, sleepiness and lower motivation to study, all of which affect learning and leads to poor academic performance [7,19,20,21]. In fact, literature shows that adolescents’ poor sleep quality, reduced sleep time, and excessive sleepiness is related to lower grades [4,22]. These consequences are expected to affect the adolescent’s development, growth, and functioning [6,7,12,23]. In sum, sleep insufficiency is a problem with major repercussions to adolescents’ lives.

Among the many possible explanations for sleep insufficiency, one possibility is simply that people are going to bed late, i.e., are delaying going to bed, and, consequently, sleep less than recommended or necessary. This delay or postponement can be observed from many perspectives and, in the present study, it will be framed in a behavioral perspective, specifically, through the lens of procrastination.

Procrastination refers to the act of “voluntarily delay an intended course of action despite expecting to be worse off for the delay” [24] (p. 66), and usually implies engaging in activities that are more appealing than, and likely to replace, the task to be completed [24]. This behavior is pervasive across life domains and highly prevalent. For example, Ferrari, Diaz-Morales, O’Callaghan, Díaz, and Argumedo [25] reported that 20%–25% of the general population procrastinates, while 70% of university students consider themselves academic procrastinators [26,27]. Literature has been working to uncover the antecedents of academic procrastination by adopting different perspectives [24,28] and individual differences (e.g., the Big Five [29]); characteristics of the task (e.g., aversive tasks are more often postponed [24]); demographic characteristics (e.g., age, sex [24,30]); and motivational and volitional aspects (e.g., fear of failure [31]; inability to regulate one’s behavior [32]). The detrimental impact of academic procrastination on school achievement and overall academic performance [33,34], as well as in other spheres of an individual’s life (e.g., mental stress, anxiety, overall low life satisfaction, and poor physical health [35,36]) is substantial and well documented. This scenario has reinforced researchers’ efforts to look at procrastination beyond the academic domain [30,32,36]. Procrastination in the health area illustrates such a shift in researchers’ attention [36], as procrastination may affect the well-being and health of individuals. For example, procrastination is linked to depression, anxiety [37], and distress [38]. Moreover, many people delay exercising or eating healthy [36]; likewise, individuals can also procrastinate their time to go to bed which may compromise a good night of sleep. 

The concept of Bedtime Procrastination was introduced by Kroese and colleagues [39] and was defined as the act of “going to bed later than intended, without having external reasons for doing so” [40] (p. 854). This procrastination behavior requires a delay (“going to bed later than intended”), the absence of a valid reason that could explain the delay (e.g., being sick) and, finally, knowing that their actions will result in negative consequences [41,42]. Procrastination usually involves the delay of a task that individuals find aversive (e.g., writing a report or doing the laundry). However, sleeping is not a task that most people consider aversive, on the contrary, sleeping scores above average in self-reported enjoyment, suggesting individuals do not avoid sleeping on purpose [43]. Bedtime Procrastination is a relatively new concept; thus, literature on this topic is scarce. Nevertheless, so far, research has shown that Bedtime Procrastination is negatively associated with trait self-regulation [39,44], hours of sleep [44,45], and self-control [46] and positively with perceived insufficient sleep [39,44,45], daily fatigue [44], and general procrastination [39]. The role of individual circadian rhythms on individuals’ tendency to procrastinate their bedtime is yet to be fully understood. For example, Kuhnel and colleagues [47] describe that individuals with later chronotypes (or eveningness) seem to report more Bedtime Procrastination. Conversely, Kadzikowska-Wrzosek [44] reported that Bedtime Procrastination was negatively associated with morningness but, unexpectedly, Bedtime Procrastination was not positively associated with eveningness. Nevertheless, the possibility of a biological predisposition to procrastinate before going to bed does not rule out the role of self-regulation in managing this behavior [48].

### Purpose of the Study

Literature on Sleep Procrastination has typically focused on behaviors preceding bedtime (Bedtime Procrastination) [40]. However, there is also the possibility that individuals delay going to sleep by engaging in activities while they are already in bed, which we have coined as While-in-Bed Procrastination. Nowadays, individuals have open access to a vast diversity of sources of entertainment with the potential of keeping them up all night [40]. From cellphones to computers and television, the access to different distractions is unlimited, and the enrolment in distractions may cause the postponement of the desired hour to sleep. Additionally, these electronic devices can be, and are being, used everywhere, including in adolescents’ rooms and beds, meaning that electronic media use can be considered a disruptor of sleep [11]. In fact, research shows that the presence of media technology in children’s rooms is predictive of shorter sleep duration and poorer sleeping habits [49]. Worryingly, a recent study developed by Vernon, Modecki, and Barber [50] showed that the use of mobile phones when lights were off increased during a three-year period. Altogether, this suggests individuals can lay in their beds and scroll on their social networks, watch movies on TV, or YouTube videos on their smartphones for hours, losing control of their sleep schedule. That is, individuals may accomplish their desired bedtime but fail to sleep the desired number of hours; in sum, people may go to bed on time but fail to sleep on time.

Acknowledging extant research, the main goal of this exploratory study was to explore whether the While-in-Bed Procrastination is a novel phenomenon that adds to the Sleep Procrastination literature. Additionally, we also aimed to understand how related features, such as demographic characteristics, sleep and daily life routines, among others, may differ between these two facets of Sleep Procrastination. Thus, we studied the Sleep Procrastination phenomenon in high school students by analyzing the role of While-in-Bed Procrastination, along with the Bedtime Procrastination. Our hypothesis is that Sleep Procrastination is composed of two distinct facets: Bedtime and While-in-Bed Procrastination.

## 2. Materials and Methods

### 2.1. Participants

Six hundred and nineteen participants initiated the questionnaire and 415 completed it (completion rate of 67%). Data from 15 participants who scored an abnormal distance from other values in the sample (outliers) were excluded. From the remaining sample (*n* = 400), 139 (34.75%) were male. The mean age was 16.56 years (SD = 1.07, range 15–20). The majority (*n* = 156, 39.2%) attended the 12th grade, 129 (32.4%) the 11th grade, and 113 (28.4%) the 10th grade.

### 2.2. Instruments and Measures

#### 2.2.1. Sociodemographic Questionnaire

Demographic questions included sex, age, and school year.

#### 2.2.2. Adaptation and Development of the Questionnaires

To develop the questionnaires used in this study, a modified Delphi survey technique was used [51,52,53,54]. This method consists of an iterative approach that develops over several rounds, in a flexible and stirring way. In the several rounds, a group of experts and relevant stakeholders are invited to provide their opinion and share their knowledge and experience about a particular theme or problem. This approach allows reaching a consensus about the indicators that are being developed by the researchers. In the present study, this process was informed both by the literature and group discussions with experts who work with adolescents on a daily basis (i.e., educational psychologists, educators, and clinicians). Two meetings were held between the researchers and experts, with each lasting approximately 3 h.

The first meeting was an open planned discussion, in which all interveners had the opportunity to share their opinions, thoughts, experience, and expertise on the topic. This group meeting involved three main phases: introduction, main discussion, and systematization. During the introduction, researchers presented the purpose of the study and relevant literature on the topic, as well as the purpose of the group meeting. It was presented the Bedtime Procrastination Scale [39] and relevant variables related with the phenomenon already identified in the literature (e.g., hours of sleep, perception of tiredness during the day [44]), as well as literature on adolescents’ use of electronic devices in their rooms and beds [11]. The main discussion focused on the sharing of experts’ thoughts on the literature presented in light of the goal of the meeting, i.e., develop a questionnaire to evaluate Sleep Procrastination while-in-bed and relevant contextual variables. The systematization phase served to summarize the main points that emerged during the main discussion. This meeting provided researchers with the material and information to elaborate questions to devise the two questionnaires, one pertaining While-in-Bed Procrastination and the other regarding contextual variables of the adolescent.

The second meeting was held to present the draft of the questionnaires to the experts to check whether the information presented was appropriate, covered all aspects discussed, and was deemed relevant for the purpose of the study. This group meeting involved three main phases: review, presentation and discussion of the questionnaires, and closure. During the review, the main conclusions of the first meeting were enumerated. The presentation and discussion of the questionnaires phase focused on assessing the appropriateness of the questions and language, and whether all aspects enumerated were included. Lastly, the closure phase served to draft the final version of the questionnaire.

##### Bedtime Procrastination Scale

Bedtime Procrastination was evaluated through the Bedtime Procrastination Scale developed by Kroese and colleagues [39]. This is a nine-item instrument (e.g., “I go to bed later than I had intended”) and items were answered in a five-point Likert-like scale from 1 (never) to 5 (always). Total scores ranged between 9 and 45 with higher scores indicating more engagement in Bedtime Procrastination (Cronbach’s alpha = 0.92). The original questionnaire was developed in adults, and Portuguese version was adapted for the adolescent population. The final questionnaire (adapted version) was composed of eight items (item “If it is time to turn off the lights at night I do it immediately” did not saturate), with total scores ranging between 8 and 40, and a Cronbach’s alpha of 0.85. The appropriateness of the factor analysis was supported by Bartlett’s test of sphericity, which provides an indicator of the strength of the relationships among variables. Bartlett’s test of sphericity χ2 (28) = 1112,525.47, *p* < 0.001, indicated that the correlation structure is an adequate for factor analyses. Moreover, we conducted the Kaiser–Meyer–Olkin (KMO = 0.875) and found that the sampling was adequate for the analysis [55,56]. To explore the factorial structure of the Bedtime Procrastination Scale, the eight items of the instrument were subjected to an exploratory factor analysis with oblique rotation (oblimin). The maximum likelihood factor analysis with a cut-off point of 0.50 and the Kaiser’s criterion of eigenvalues greater than 1 yielded a one-factor solution as the best fit for the data, accounting for 49.35% of the variance.

##### While-in-Bed Procrastination Scale

To assess whether adolescents delay sleeping when they are already in bed, and to learn which activities they engage in before going to sleep, the While-in-Bed Procrastination Scale was developed. This is a seven-item instrument (e.g., “In bed, before I fall asleep, I watch videos on Youtube” or “In bed, before I fall asleep, I eat snacks (cookies, cereals, milk, chips, chocolate)”), and items are answered on a five-point Likert-like scale from 1 (almost never) to 5 (almost always). Total scores ranged from 7 to 35 with higher scores indicating more While-in-Bed Procrastination (Cronbach’s alpha = 0.732). We followed procedures similar to those run in the previous factor analysis. Bartlett’s test of sphericity χ2 (21) = 441,909, *p* < 0.001, indicating that correlation structure is adequate for factor analyses. In addition, the Kaiser–Meyer–Olkin measure (KMO = 0.805) verified the sampling adequacy for the analysis [55,56]. Finally, an exploratory factor analysis with oblique rotation (oblimin) was run to examine the factorial structure of the While-in-Bed Procrastination Scale (seven items). The maximum likelihood factor analysis with a cut-off point of 0.50 and the Kaiser’s criterion of eigenvalues greater than 1 yielded a one-factor solution as the best fit for the data, accounting for 38.49% of the variance.

##### Contextual Variables

The inclusion of sleep-related variables was discussed with the panel of experts in the first meeting. The aim was to gather information on the environment and circumstances that the participants live in; these data were expected to provide a better understanding of the Sleep Procrastination phenomenon. The selection of the variables and their metrics was informed by the experts’ inputs and by authors’ experience while working with adolescents in educational topics. Finally, a set of variables related to sleep descriptives, family context, and daily routines was included in the research protocol.

Sleep Descriptives: questions about sleep habits and tiredness were included in the questionnaire. Participants were asked to indicate, by selecting one of the options, their usual waking time (before 7:00 a.m.; between 7:00 a.m. and 7:59 a.m.; between 8:00 a.m. and 8:59 a.m.; after 9:00 a.m.) and their desired hour to fall asleep (this was an open question coded into: 9:00 p.m. to 9:59 p.m.; 10:00 p.m. to 10:59 p.m.; 11:00 p.m. to 11:59 p.m.; 12:00 a.m. to 12:59 a.m.; 1:00 a.m. to 3:00 a.m.). Furthermore, participants were asked to quantify the amount of sleep they have per night on average (this was an open question coded into: Less than 5 h; 5–6 h; 6–7 h; 7–8 h; 8–9 h; 9–10 h) and whether they usually feel tired during the day (Almost never, Sometimes, Almost always).

Regarding Family Context, participants were asked about their parents’ usual time for arriving home (5:00 p.m.; Between 5:00 p.m. and 7:00 p.m.; Between 7:00 p.m. and 9:00 p.m.; After 9:00 p.m.), the number of siblings (0; 1; 2; 3; 4 or more), and whether they share their bedroom with someone (e.g., sibling).

Lastly, Daily Routine questions were added. Participants were asked about their usual dinnertime (around 7:00 p.m.; around 7:00 p.m. and 8:00 p.m.; after 9:00 p.m.), their school starting time (Between 8:00 a.m. and 10:00 a.m.; Between 10:00 a.m. and 11:00 a.m.; In the afternoon), and, finally, if they practice any sports and if affirmative, at what time they usually finish (Before 3:00 p.m.; 3:00 p.m. to 3:59 p.m.; 4:00 p.m. to 4:59 p.m.; 5:00 p.m. to 5:59 p.m.; 6:00 p.m. to 6:59 p.m.; 7:00 p.m. to 7:59 p.m.; 8:00 p.m. to 8:59 p.m.; 9:00 p.m. to 9:59 p.m.; 10:00 p.m. to 10:59 p.m.; 11:00 p.m. to 11:59 p.m.).

Additionally, participants were asked to position themselves regarding the following question, by selecting one of the options: “Do you consider that you procrastinate (delay the hour at which you go to sleep): before going to bed or when you are already in bed?”.

### 2.3. Procedure

The present study is part of a research project that has been approved by the University of Minho Ethics Committee for Research in Social and Human Sciences (CEICSH) (CEICSH 032/2019).

The study is cross-sectional in design, and data were collected from January through March 2019. The inclusion criterion was that respondents should be enrolled in and attending high school. The survey was administered in Portuguese language only and took approximately 10 min to complete. The link to the survey was posted on social media (Instagram, Facebook) and distributed through personal contacts (e.g., text-messages and emails, soliciting the sharing of the survey with family members and friends that could be in the appropriate age for completing). Recruitment from social media was through the following strategy: authors shared the link in their personal feed urging friends and followers to participate in the study, in the case of being students enrolled in high school, or shared the link with their friends and family members that could be in the target population. No information regarding geographic or cultural make-up of the participants was recorded. An informed consent was obtained from the participants prior to their participation through a “yes or no” question on the first page of the survey. Participation in the study was voluntary, anonymous, and unpaid. Participants who gave their consent to participate filled in the electronic questionnaire that assessed sociodemographic questions, contextual variables, Bedtime Procrastination, and While-in-Bed Procrastination, in this specific order.

### 2.4. Data Analysis

The questionnaire included three different sections for analyses, the sociodemographic questions, the contextual variables, and the Bedtime Procrastination and While-in-Bed Procrastination scales. Contextual variables were collected through a different format of answers. To facilitate the analyses, interval variables were transformed to ordinal variables. For example, the question “Usually, your waking time is around” was categorized as 1 (before 7:00 a.m.), 2 (between 7:00 a.m. and 8:00 a.m.), 3 (between 8:00 a.m. and 9:00 a.m.), and 4 (after 9:00 a.m.). Questions with an open-format answer, for example “What time is your sport over?”, were grouped according to participants’ answers. Answers were categorized as 1 (before 3:00 p.m.), 2 (3:00 p.m. to 3:59 p.m.), 3 (4:00 p.m. to 4:59 p.m.), 4 (5:00 p.m. to 5:59 p.m.), 5 (6:00 p.m. to 6:59 p.m.), 6 (7:00 p.m. to 7:59 p.m.), 7 (8:00 p.m. to 8:59 p.m.), 8 (9:00 p.m. to 9:59 p.m.), and 9 (10:00 p.m. to 10:59 p.m.). Answers regarding amount of hours slept per night and number of siblings were not transformed.

The dataset was collected, treated, and analyzed using the SPSS^®^ version 24 (IBM Corporation, Armonk NY, USA) for Windows^®^. Descriptive statistics and frequency analyses were conducted for all variables in the study. Factorial analysis including the KMO and Bartlett Test, the Communalities test, the total explained variance test, the component matrix, and the reliability test were conducted on both scales. Pearson’s correlation was conducted between the Bedtime Procrastination Scale and the While-in-Bed Procrastination Scale. Lastly, multiple ANOVAs were conducted between sociodemographic variables, the procrastination scales, and the contextual variables. Gabriel’s Post-Hoc Test was used for a pairwise comparison.

## 3. Results

### 3.1. Descriptive Statistics of Contextual Variables

Half of the participants (53.2%) reported sleeping seven hours or less per night, while only 10.3% of the sample reported sleeping the recommended number of hours (M = 7.4, SD = 0.9). There was a relatively strong negative correlation between Bedtime Procrastination and the number of sleeping hours (r = −0.403; *p* < 0.005), but no correlation was found between While-in-Bed Procrastination and number of sleeping hours (r = −0.057; n.s).

Additionally, 58.8% reported feeling tired sometimes during the day, while 22.8% reported feeling tired “almost always”. More than half of the sample reported that their parents arrive home between 5:00 p.m. and 7:00 p.m. Regarding the number of siblings, the majority (62.3%) reported to have one sibling, while the minority (1.1%) reported to have four or more siblings. Regarding dinnertime, 68% of the participants reported to have dinner between 7:00 p.m. and 8:00 p.m., while only 5.3% of the sample reported to have dinner at 7:00 p.m. Moreover, 68% of the sample has classes starting between 8:00 a.m. and 9:00 a.m., and 85.5% do not share their rooms. Relatively to physical activity, 56.8% reported not playing sports, against 43.2% who practice sports at least once a week. Among the participants who practice sports, 12.1% finish before 06:00 pm, 47.1% between 6:00 p.m. and 8:59 p.m., and finally, 40.8% after 9:00 p.m. Finally, participants were asked whether they procrastinate before going to bed or while-in-bed. The majority (59.5%) reported to procrastinate while-in-bed, whereas the remaining reported to procrastinate before going to bed.

### 3.2. Test of Association between Bedtime and While-in-Bed Procrastination

Sleep Procrastination was evaluated via two questionnaires: The Bedtime Procrastination, developed by Kroese and colleagues [39], and the While-in-Bed Procrastination, developed for the present study. The Portuguese version of the Bedtime Procrastination scale has eight items with high interitem reliability (Cronbach’s α = 0.85). Table 1 displays descriptive statistics for each item, with a mean score for this scale of 3.2 (SD = 0.9).

Regarding the While-in-Bed Procrastination scale, it comprises seven items with a moderate interitem reliability (Cronbach’s α = 0.73). Table 2 displays descriptive statistics for each item, with a mean score for this scale of 2.9 (SD = 0.9). The most performed behaviors were “send texts, make video calls/calls” and “watch videos on YouTube”, while the least performed behavior was “eat snacks” (cookies, cereals, milk, chips, chocolate).

Pearson’s correlation was calculated between Bedtime Procrastination and While-in-Bed Procrastination. This allows testing whether the scales are measuring the same construct, if they are highly correlated, or whether they are measuring different constructs, if they are poorly or not correlated. Data show that the correlation between scales is low (r = 0.158 **), which supports the contention that Sleep Procrastination may be composed of two facets: Bedtime Procrastination and While-in-Bed Procrastination.

### 3.3. Analysis of Variance

Analyses of Variance were performed for some contextual and sociodemographic variables with Bedtime Procrastination and While-in-Bed Procrastination. Concerning Bedtime Procrastination, results show that there were significant differences in Bedtime Procrastination regarding waking time and dinner time: F(3.396) = 4.63, *p* < 0.005, η_p_^2^ = 0.184 and F(2.397) = 7.13, *p* < 0.005, η_p_^2^ = 0.186, respectively. Gabriel’s Post-Hoc Test revealed that participants reporting waking up after 9:00 a.m. reported more Bedtime Procrastination, compared to participants reporting waking up before 7:00 a.m. and between 7:00 a.m. and 8:00 a.m. Regarding dinnertime, participants reporting having dinner after 9:00 p.m. reported more Bedtime Procrastination, compared to participants reporting having dinner at 7:00 p.m. and between 7:00 p.m. and 8:00 p.m.

In relation to While-in-Bed Procrastination, the scenario alters. Results showed that there were significant differences regarding sex: F(1, 398) = 11.73, *p* < 0.005, η_p_^2^ = 0.170, desired sleep time, F(4, 389) = 5.999, *p* < 0.001, η_p_^2^ = 0.241,and dinner time, F(2, 397) = 6.181, *p* < 0.005, η_p_^2^ = 0.174. Gabriel’s Post-Hoc Test showed that male participants reported more While-in-Bed Procrastination compared to female participants. Regarding desired sleep time, adolescents who reported wanting to sleep between 1:00 a.m. and 3:00 a.m. reported more While-in-Bed Procrastination than adolescents who reported wanting to sleep between 9:00 p.m. and 9:59 p.m., 10:00 p.m. and 10:59 p.m., 11:00 p.m. and 11:59 p.m. and, and 12:00 a.m. and 00:59 a.m. Lastly, the Post-Hoc Test regarding the variable Dinnertime showed that there were differences between the group of participants who reported having dinner at 7:00 p.m. and the other two groups. Accordingly, participants who reported having dinner at 7:00 p.m. tended to engage more in While-in-Bed Procrastination than participants who reported having dinner between 7:00 p.m. and 8:00 p.m. and after 9:00 p.m.

## 4. Discussion

The goal of the present study was to examine whether the Sleep Procrastination phenomenon is composed of two distinct facets: Bedtime and While-in-Bed procrastination and particularly, to analyze the role of While-in-Bed Procrastination, along with the Bedtime Procrastination, in the Sleep Procrastination phenomenon in high school students. The key findings are that Sleep Procrastination may be composed of the two facets of Bedtime Procrastination and While-in-Bed Procrastination, and that each may be associated with different variables or characteristics of the individual. This study will contribute to new research avenues on the Sleep Procrastination domain, and to inform tailored interventions designed to tackle Sleep Procrastination.

Sleep plays a vital role in our health. Sleeping the recommended number of hours has benefits to cognition, mental and physical health, and conveys wellbeing [57,58,59,60,61]. The current study found that adolescents, who need a good sleep hygiene for their normative development, are sleeping two hours less per night than the recommended number of hours. This finding, despite worrying, is consistent with previous literature [62,63]. Amidst this scenario, the purpose of this paper was to explore the possibility that sleep insufficiency may be due to procrastination of sleep. Particularly, we looked at Sleep Procrastination as a broader phenomenon, aiming to examine whether there is evidence that this construct consists of two facets: Bedtime Procrastination and While-in-Bed Procrastination.

To measure Bedtime Procrastination, the Bedtime Procrastination Scale, developed by Kroese and colleagues [39], was adapted and validated for the Portuguese population. The authors provided additional information about the scale results (frequencies of the items), and it can be concluded that the Portuguese version follows the same tendency as the original scale. The means of the items were particularly similar (the maximum variation was one point in one item) as well as the general means of the scale. Regarding the concept of While-in-Bed Procrastination, we developed a scale to capture this phenomenon. The correlation between both scales was low (*p* = 0.158 **), suggesting that these instruments are measuring different phenomena, thus supporting our assertion that Sleep Procrastination may comprise two aspects: procrastination behaviors before going to bed and after going to bed.

Interestingly, the behaviors that individuals reported engaging more with while in bed were watching videos on YouTube, watching movies, listening to music, and sending texts. These findings are consistent with those reported by the National Sleep Foundation [22]. This report indicated that all adolescents used at least one electronic device in bed, particularly: 57% television, 90% music players, 43% computers, and 64% phones. Additionally, the study by Calamaro, Mason, and Ratcliffe [64] reported that adolescents were sending texts, making phone calls, playing computer games or were online after 9:00 p.m. Similarly, the study by Van den Bulck [65] using a Belgian sample reported that 62% of adolescents used their phone in bed, with the lights off, and consequently, reported more tiredness during the following day.

Lastly, the present study allowed examining differences in contextual variables as a function of type of procrastination, Bedtime or While-in-bed. Whereas Bedtime Procrastination was related to the context variables waking time and dinnertime, While-in-Bed Procrastination was linked to sex, desired time to sleep, and dinnertime. This pattern seems to indicate three essential aspects: sex, ambient control, and sleep goals. More specifically, it seems that sex, ambient control (dinnertime), and sleep goals (desired sleep and wake hour) predicted distinctly the facet of Sleep procrastination (Bedtime or While-in-Bed procrastination). According to the literature, men show lower self-regulatory skills than women and, therefore, engage further in procrastination [66,67].

Present results support the assertion that Sleep Procrastination has two facets and that these facets not only differ in their nature but also may encompass different aspects. Procrastinating going to bed contemplates contextual variables (e.g., individuals who wake up early tend to procrastinate their bedtime less) and has a strong negative correlation with number of hours of sleep. Conversely, procrastinating sleeping while already in bed encompasses not only contextual topics but also motivation-related aspects such as goal-setting (i.e., desired time to sleep) and is not correlated with number of hours of sleep. It seems that the individuals in the latter group may interpret being already in bed as having accomplished their sleeping goals (e.g., to sleep 8 h per night). A recent qualitative study [68] has provided some insight on the explanations people provide for delaying going to bed. Particularly, deliberate procrastination, when the delay is intentional and often associated with the completion of tasks (e.g., cleaning); mindless procrastination, when the delay is the result of distraction and inattention (e.g., engaged in an immersive activity); and strategic delay, when the delay is associated with the belief that individuals will fail to fall asleep if they go to bed earlier. Considering the distinct nature of the two facets of Sleep Procrastination, there is the possibility that the perceptions and interpretations of why individuals procrastinate while-in-bed are distinct from the reasons highlighted in that prior study. Indubitably, more research into this topic is necessary to shed some light over this phenomenon.

### Limitations and Future Studies

Present results encompass extreme importance, the first analyzing Sleep Procrastination as having two facets—Bedtime Procrastination and While-in-Bed Procrastination. Our novel findings are likely to disclose new research avenues and contribute to deepening our understanding on sleep insufficiency. Nevertheless, the current research has methodological constraints that should be considered when interpreting the results. First, it is important to emphasize that our While-in-Bed Procrastination Scale has not been validated in any other samples and further psychometric validation is needed. In this regard, it becomes pressing to assess this scale in samples other than adolescents, as one cannot disregard the role that chronotypes may play in the Sleep Procrastination process. Additionally, the variance explained by the one factor solution within our scale is relatively low; thus, this limitation must be acknowledged. In addition, the reader should bear in mind the correlational nature of this study, which does not provide evidence about the causality of the effects.

Second, the scope of both procrastination scales differs. Specifically, the Bedtime Procrastination Scale asks about intentions and specific behavior routines but does not include object-related activities (e.g., smartphone use, gaming, reading). In contrast, the While-in-Bed Procrastination Scale asks about specific object-related activities and does not include aspects related to intentions and specific behavior routines (e.g., thinking about what happened during the day, planning the next day, worrying about one’s problems). Thus, future studies may consider extending the scope of both Bedtime and While-in-Bed Procrastination scales to accommodate these aspects. Additionally, it could be important to understand how much time individuals engage in these activities before and after going to bed, and whether this is a daily habit or a sporadic behavior.

Third, data were collected online through a self-report questionnaire, and the formulation of some questions (e.g., “Do you consider that you procrastinate (delay the hour at which you go to sleep): before going to bed or when you are already in bed?”) may have limited and biased participant answers. Thus, we suggest future studies to apply a momentary form of evaluation (e.g., sleep diaries), which would add additional validity to the research. Moreover, considering the small sample recruited, the possible sampling bias (e.g., personal contacts options for recruitment), and the exploratory nature of the study, it is necessary to conduct further large-scale studies to confirm the findings. Nonetheless, we have no reasons to believe that these limitations bring prejudice to the study, since the reliability of the measures was high.

Taking into account the new research possibilities brought by this new conceptualization of Sleep Procrastination, future research may consider further studying the underlying mechanisms of sleep insufficiency in general and of Sleep Procrastination in particular. For example, it could be interesting to explore the variability of Sleep Procrastination prospectively and learn the effects of infrequent vs. chronic Sleep Procrastination. Additionally, it would be important to understand how different Sleep Procrastinators procrastinate in other life domains (e.g., exercise, healthy eating) and how this may affect life aspects such as academic performance. Lastly, conceiving and researching Sleep Procrastination including these two facets may contribute to informing educators and practitioners on more adjusted interventions to the procrastination behaviors individuals engage in.

## 5. Conclusions

To the best of our knowledge, this is the first study to undertake a different direction in Sleep Procrastination, adding a new perspective to the literature. The results suggest that assessing Sleep Procrastination solely focused on procrastinating behaviors before going to bed is not enough to fully understand the phenomenon. Delaying sleep while-in-bed is a common reality and needs to be acknowledged as part of the Sleep Procrastination process. Hence, this study makes an original contribution in several important aspects. As described above, sleep insufficiency is a subject of extreme importance and affects a significant portion of the general population; so, expectedly, this study will contribute to its improvement.

## Figures and Tables

**Table 1:** Bedtime Procrastination Scale frequencies.

Item	Almost Never	Rarely	Neutral	Frequently	Almost Always	Mean *(SD)*
I go to bed later than I had intended	32 (8%)	78 (19.5%)	85 (21.3%)	124 (31%)	81 (20.3%)	3.36 *(1.23)*
I go to bed early if I have to get up early in the morning (R)	58 (15.5%)	98 (24.5%)	120 (30%)	94 (23.5%)	30 (7.5%)	2.85 *(1.16)*
Often I am still doing other things when it is time to go to bed.	20 (5%)	48 (12%)	64 (16%)	170 (42.5%)	98 (24.5%)	3.70 *(1.12)*
I easily get distracted by things when I actually would like to go to bed.	43 (10.8%)	60 (15%)	95 (23.8%)	129 (32.3%)	73 (18.3%)	3.32 *(1.24)*
I do not go to bed on time.	43 (10.8%)	61 (15.3%)	104 (26%)	113 (28.2%)	79 (19.8%)	3.31 *(1.25)*
I have a regular bedtime which I keep to (R).	42 (10.5%)	94 (23.5%)	89 (22.3%)	84 (21%)	91 (22.8%)	3.22 *(1.32)*
I want to go to bed on time but I just don’t.	76 (19%)	89 (22.3%)	85 (21.3%)	89 (22.3%)	61 (15.3%)	2.93 (*1.347)*
I can easily stop with my activities when it is time to go to bed (R).	30 (7.5%)	81 (20.3%)	118 (29.5%)	107 (26.8%)	64 (16%)	3.24 *(1.17)*

**Table 2:** While-in-Bed Procrastination Scale frequencies.

Item	Almost Never	Rarely	Neutral	Frequently	Almost Always	Mean (*SD*)
*While in bed, before I fall asleep I…*						
…Watch videos on YouTube	69 (17.3%)	58 (14.5%)	46 (11.5%)	129 (32.3%)	98 (24.5%)	3.32 *(1.43)*
…Watch TV	142 (35.5%)	82 (20.5%)	63 (15.8%)	59 (14.8%)	54 (13.5%)	2.5 *(1.44)*
…Watch movies and series	89 (22.3%)	67 (16.8%)	66 (16.5%)	100 (25%)	78 (19.5%)	3.03 *(1.45)*
…Listen to music	76 (19%)	66 (16.5%)	48 (12%)	100 (25%)	110 (27.5%)	3.26 *(1.49)*
…Send texts, make video calls/calls	59 (14.8%)	46 (11.5%)	60 (15%)	110 (27.5%)	125 (31.3%)	3.49 *(1.41)*
…Play games (tablet, computer, mobile phone)	158 (39.5%)	69 (17.3%)	68 (17%)	55 (13.8%)	50 (12.5%)	2.43 *(1.44)*
…Eat snacks (cookies, cereals, milk, chips, chocolate)	185 (46.3%)	68 (17%)	58 (14.5%)	52 (13%)	37 (9.3%)	2.22 *(1.38)*
